# The Optic Nerve as a New Diagnostic Frontier in Multiple Sclerosis: How the 2024 McDonald Criteria Leverage Multimodal Evaluation for Earlier Diagnosis

**DOI:** 10.7759/cureus.97579

**Published:** 2025-11-23

**Authors:** Nikolaos Mitsoudis, Ioannis Nikolakakis, Virginia Giantzi, Demetrios Pirounides, Christos Bakirtzis

**Affiliations:** 1 Second Department of Neurology, Aristotle University of Thessaloniki, AHEPA Hospital, Thessaloniki, GRC; 2 Department of Ophthalmology, Aristotle University of Thessaloniki, AHEPA Hospital, Thessaloniki, GRC

**Keywords:** mcdonald criteria, multiple sclerosis, optical coherence tomography, optic nerve, optic neuritis

## Abstract

The revised 2024 McDonald criteria recognize the optic nerve as a fifth topographical location for dissemination in space (DIS), raising critical questions regarding its diagnostic utility and technical requirements. To analyze this new inclusion, we provide a focused review and illustrate its clinical significance through a case series of three patients where optic nerve assessment was critical for diagnosis. We analyze the operationalization of key diagnostic tools, including optical coherence tomography (OCT) (defining retinal nerve fiber and ganglion cell layer thinning thresholds and inter-eye asymmetry, assessed outside acute neuritis), visual evoked potentials (VEPs) (P100 latency criteria), and 3T orbital magnetic resonance imaging (MRI) (using fat-suppressed T2/short-tau inversion recovery (STIR) and post-gadolinium sequences). Furthermore, we specify how dissemination in time (DIT) was met (presence of CSF-specific oligoclonal bands or new T2/gadolinium-enhancing lesions) and how key mimics (e.g., myelin oligodendrocyte glycoprotein antibody disease (MOGAD), neuromyelitis optica spectrum disorder (NMOSD), ischemic/hereditary neuropathies) were excluded via antibody testing and clinical/laboratory findings. The analysis suggests that the 2024 multiple sclerosis (MS) criteria, by recognizing the optic nerve, may enhance diagnostic sensitivity. In our cohort, multimodal optic nerve assessment was essential to meet DIS criteria, successfully expediting the MS diagnosis and treatment initiation. This multimodal approach also highlights the optic nerve's potential as a source for quantitative biomarkers and a target for future neuroprotective strategies. We conclude that including the optic nerve as a DIS site represents a major diagnostic advance. The presented cases underscore the practical application of the 2024 criteria, stressing the importance of comprehensive evaluation, but also highlighting the critical need for careful exclusion of mimics, vigilance to avoid overdiagnosis, and the necessity of standardized acquisition protocols and robust normative databases for generalizing this approach.

## Introduction

The optic nerve originates from the brain and is enveloped by central nervous system (CNS) meninges, forming a continuous subarachnoid space [1]. Its axons are myelinated by oligodendrocytes, establishing a blood-brain barrier that is incomplete at the optic nerve head. This unique anatomy creates a region vulnerable to inflammatory demyelination and conduction block.

Optic neuritis (ON) is an inflammatory demyelinating disorder of the optic nerve, usually causing unilateral, subacute visual loss with retro-orbital pain worsened by eye movement [1]. Typical features include reduced visual acuity, impaired colour vision, central scotoma, and relative afferent pupillary defect, with vision worsening over days and recovering over weeks to months.

Multiple sclerosis (MS) is a leading cause of non-traumatic neurological disability, presenting a significant diagnostic challenge due to its heterogeneous clinical course. In MS, ON is a common initial presentation, affecting up to 50% of patients during their disease course [2]. Historically recognized as a window into CNS demyelination by pioneers like Charcot [3,4], its diagnostic value was progressively confirmed. The development of visual evoked potentials (VEPs) provided the first biomarker for subclinical lesions, while magnetic resonance imaging (MRI) later allowed in vivo visualization of optic nerve lesions, correlating structural and functional abnormalities [5]. This long-standing clinical and technological recognition culminated in the 2024 McDonald criteria [6,7].

Over the past six decades, MS diagnostic criteria have evolved from clinical observation to incorporating cerebrospinal fluid (CSF) analysis, VEPs, and MRI to assess dissemination in time (DIT) and dissemination in space (DIS) [8]. The 2024 revision represents a major advance by formally recognizing the optic nerve as a fifth anatomical site for DIS. This change endorses the use of optical coherence tomography (OCT), VEPs, and orbital MRI to provide objective, quantifiable evidence of optic nerve involvement. Addressing a key diagnostic gap, this update aims to facilitate earlier diagnosis, even in atypical presentations where patients previously may not have met DIS criteria, positioning the optic nerve as a central, measurable site in MS assessment [6].

To demonstrate the practical application of this major update, we present three consecutive clinical cases that illustrate how multimodal optic nerve assessment can expedite the diagnosis of MS under the new 2024 criteria and facilitate timely treatment.

Methods 

We report three consecutive patients with optic nerve involvement who satisfied the 2024 McDonald criteria for MS, despite not meeting earlier versions of the criteria. Patients were included if they exhibited clinical or subclinical optic nerve pathology confirmed by multimodal assessment and had supporting MRI or CSF evidence. Each patient underwent multimodal assessment, including OCT, VEPs, high-resolution brain and spinal MRI, CSF analysis, and laboratory testing to exclude mimics. We defined optic nerve involvement by VEPs based on P100 latency delays and by OCT based on pathological retinal nerve fiber layer (RNFL) or ganglion cell-inner plexiform layer (GCIPL) thinning. Cases with alternative causes of optic neuropathy, including myelin oligodendrocyte glycoprotein antibody (MOG) IgG or aquaporin-4 antibody (AQP4) IgG-associated disease, were excluded. Demographic information, clinical presentation, imaging and electrophysiological findings, and CSF results were collected for each patient. The revised 2024 McDonald criteria were applied to determine whether the combination of optic nerve involvement and other CNS findings fulfilled DIS and DIT requirements. This study was approved by the local institution’s ethical committee and was performed according to the Declaration of Helsinki and its later amendments. All subjects provided written informed consent for the publication of their medical records.

## Case presentation

We report three consecutive patients demonstrating optic nerve involvement fulfilling the 2024 McDonald criteria for multiple sclerosis. Key clinical, imaging, electrophysiological, and laboratory findings are summarized below (Table 1).

**Table 1:** Summary of Multimodal Assessment in Three Clinical Cases This table summarizes the key diagnostic findings for all three patients. Crucially, it highlights how each patient met the 2024 McDonald criteria for MS by combining evidence of a single CNS lesion (fulfilling DIS site 1) with objective, multimodal evidence of optic nerve involvement (fulfilling DIS site 2), alongside CSF-specific oligoclonal bands (fulfilling DIT). Abbreviations: OCT = Optical Coherence Tomography, VEP = Visual Evoked Potential, MRI = Magnetic Resonance Imaging, CSF = Cerebrospinal Fluid, DIS = Dissemination in Space, DIT = Dissemination in Time, MS = Multiple Sclerosis, pRNFL = Peripapillary Retinal Nerve Fiber Layer, GCIPL = Ganglion Cell-Inner Plexiform Layer, OCB = Oligoclonal Bands, IgG = Immunoglobulin G, R = Right, and L = Left.

Case	Age/Sex	Presentation	OCT Findings	VEP Findings	MRI Findings	CSF	DIS/DIT Status
1	43/F	Transient right-eye vision loss	Normal	Delayed P100 latency (R > L)	Single periventricular lesion; cervical spine normal	Type-2 OCB	DIS met; DIT supported
2	27/F	Incidental MRI; past blurred vision (L eye)	pRNFL thinning L eye; GCIPL reduction	Mild P100 delay	C1 cervical lesion; brain MRI unremarkable	Type-2 OCB	DIS/DIT met
3	29/M	Acute right-eye vision loss	Mild thinning	Delayed P100 latency (R)	Juxtacortical lesion; right optic nerve hyperintensity	Elevated IgG; CSF-restricted OCB	DIS/DIT met

Case 1

**Figure 1:**
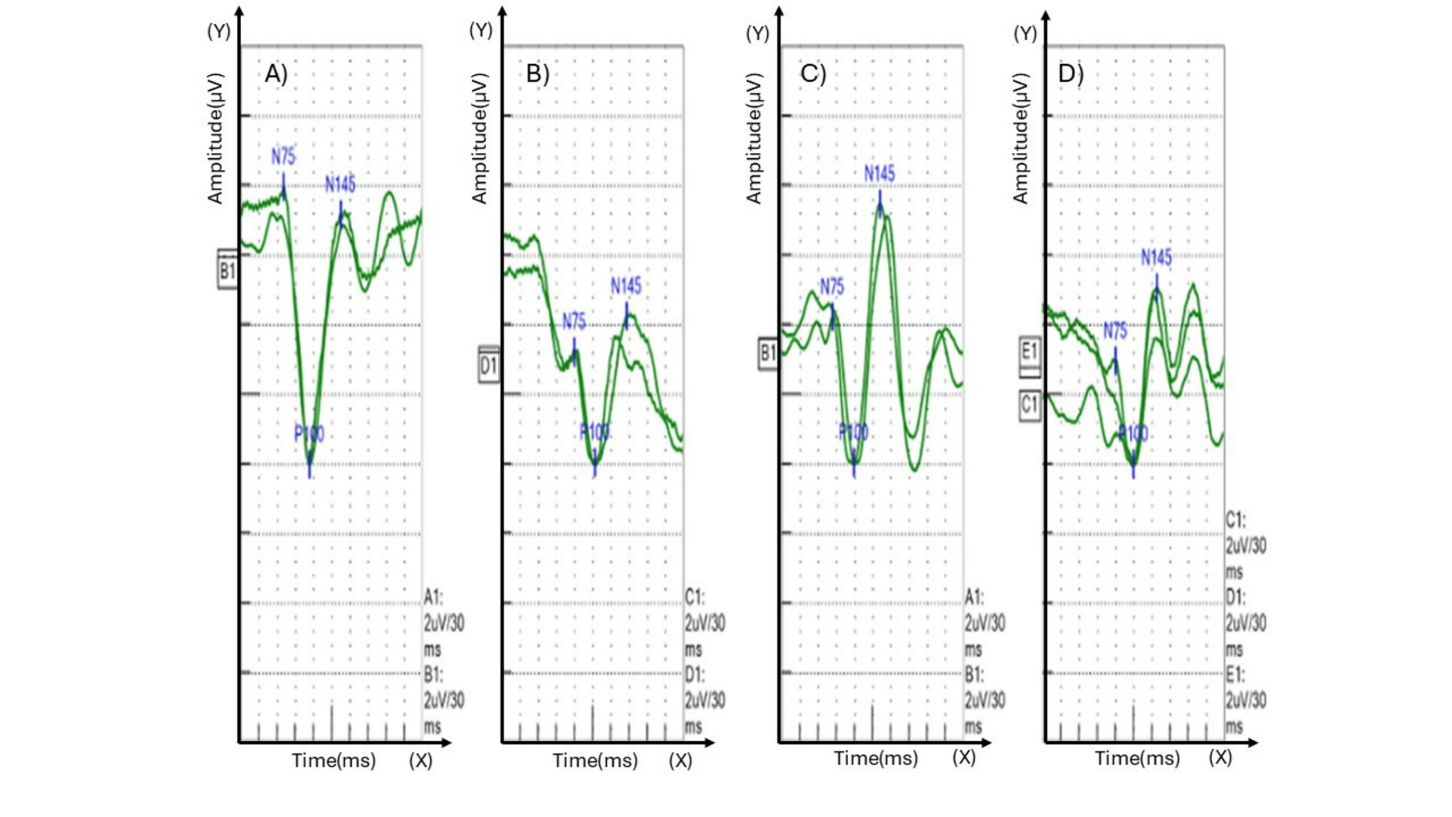
Pattern-reversal visual evoked potential (PR-VEP) recordings obtained by monocular checkerboard stimulation. PR-VEP traces demonstrate subclinical optic nerve dysfunction. Panels (A) (Left Eye, Large Checks) and (C) (Left Eye, Small Checks) show normal VEP morphology and P100 latencies. In contrast, Panels (B) (Right Eye, Large Checks) and (D) (Right Eye, Small Checks) exhibit clear pathology, characterized by significantly prolonged P100 latency and reduced amplitude (Right: 154.2 ms vs. Left: 114.0 ms for large checks). This objective electrophysiological finding of demyelinating dysfunction in the right optic nerve, despite a normal clinical exam, provided the crucial evidence of optic nerve involvement, establishing a second site for dissemination in space (DIS) under the 2024 McDonald criteria. (Axes: Y-axis represents Amplitude, scale: 2 μV/division, positive downwards; X-axis represents Time/Latency, scale: 30 ms/division).

A 43-year-old woman presented with atypical, transient vision loss in her right eye lasting approximately two hours; she reported a similar episode five years prior. Brain MRI revealed a single, ovoid, small periventricular lesion, while the cervical spine MRI was normal. CSF analysis demonstrated type-2 oligoclonal bands (OCBs). Although her ophthalmologic examination was unremarkable, VEPs showed delayed P100 latencies bilaterally (Figure 1), more pronounced on the right, exceeding the laboratory’s reference cutoff and consistent with optic nerve demyelination (Table 2). Serological tests for MOG-IgG and AQP4-IgG-associated disease were negative.

**Table 2:** Summary of pattern-reversal visual evoked potential (PR-VEP) measurements and findings This table presents objective electrophysiological data utilizing large check and small check pattern-reversal stimuli. The measurements reveal marked interocular asymmetry in both stimulus conditions. Specifically, the right eye demonstrates significantly delayed latencies (N75, P100, and N145) compared to the left eye, confirming impaired conduction time. Furthermore, the amplitude of the N75–P100 complex is substantially reduced in the right eye, resulting in a 60.1% interocular difference under large check stimulation. These findings are highly consistent with significant right optic nerve dysfunction, which supported the diagnosis of demyelination. Abbreviations: Diff. = Difference, ms = milliseconds, N75/P100/N145 = Specific waveform components of the VEP, μV = microvolts.

Stimulus Type	Side	Latency N75 (ms)	Latency P100 (ms)	Latency N145 (ms)	Amplitude N75-P100 (μV)	Interocular Diff. (%)
Large Check	Left	71.1	114.0	165.3	8.0	-
	Right	120.0	154.2	206.4	3.2	-
	Diff.	48.9	40.2	41.1	-	60.1
Small Check	Left	84.3	119.4	162.6	4.2	-
	Right	120.6	150.0	188.7	3.0	-
	Diff.	36.3	30.6	26.1	-	29.0

Case 2

**Figure 2:**
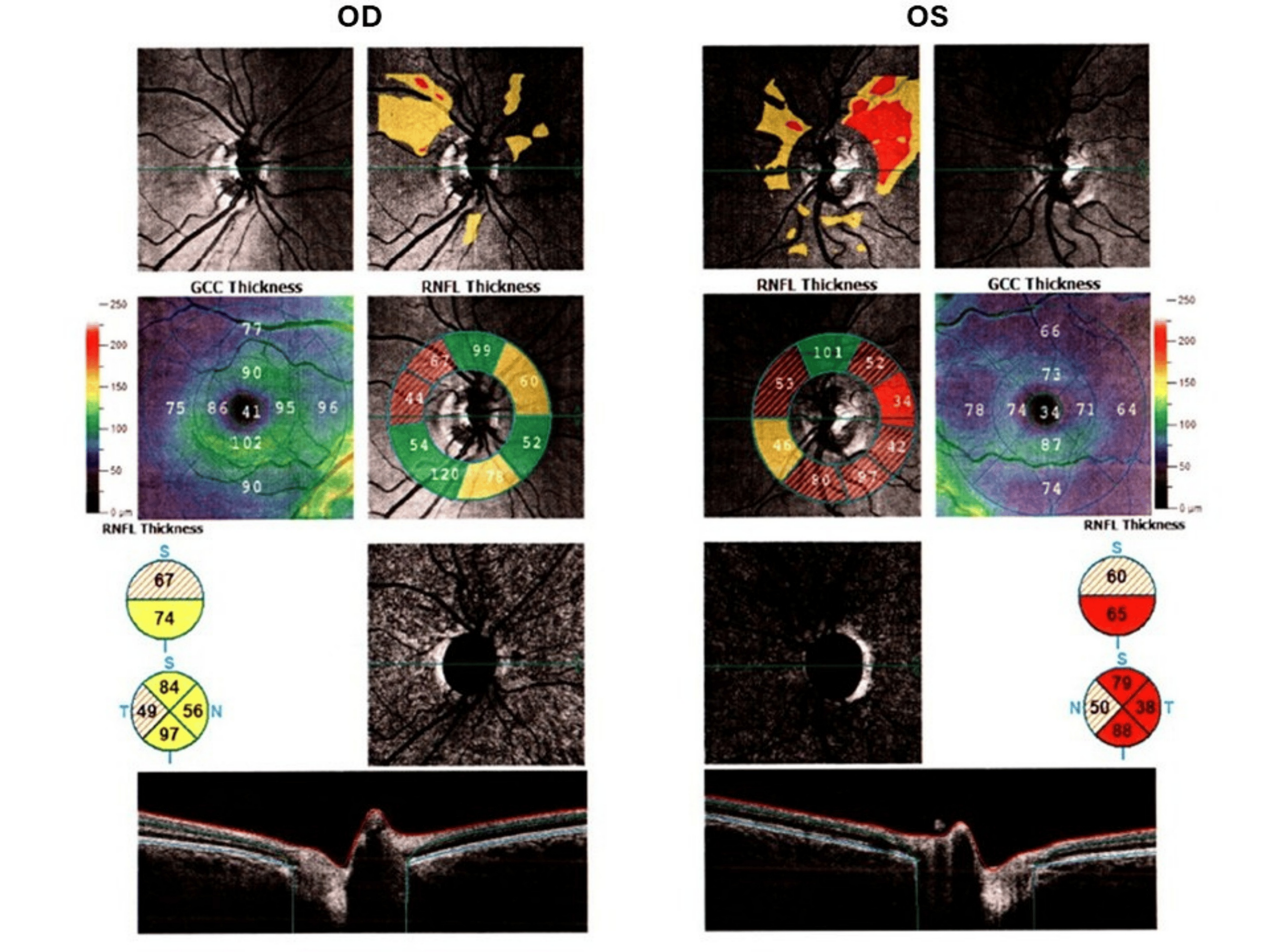
Optical coherence tomography (OCT) imaging of the right (OD) and left (OS) eyes demonstrates structural changes consistent with optic nerve damage. Right eye (OD): Retinal nerve fiber layer (RNFL) shows borderline thinning, predominantly in superior and inferior sectors (mixed green/yellow/red zones), and Ganglion cell complex (GCC) exhibits generalized reduction compared to the normative database. Left eye (OS): RNFL thinning is evident in the superior and inferior quadrants (red/yellow zones), with marked GCC thinning in central and temporal regions, suggesting ganglion cell loss. Optic disc changes suggest early-to-moderate optic nerve damage. Overall, the findings reveal bilateral RNFL and GCC thinning, more pronounced in the left eye.This objective structural damage established the optic nerve as a second dissemination in space (DIS) site.

A 27-year-old woman presented for evaluation of incidental findings on brain MRI imaging. At the time of presentation, she was asymptomatic but reported a history of a self-resolving episode of blurred vision in the left eye several years prior. Ocular examination revealed intact visual acuity; however, the left eye had a slight relative afferent pupillary defect (RAPD). Fundus examination was unremarkable. OCT showed thinning in the left peripapillary retinal nerve fiber layer (pRNFL), with clinically significant asymmetry (Figure 2), consistent with prior optic nerve injury (Table 3). Brain MRI was unrevealing, but a single demyelinating lesion was detected in the cervical spine at the C1 level. Cerebrospinal fluid analysis confirmed the presence of type 2 OCBs.

**Table 3:** Quantitative summary of optical coherence tomography (OCT) parameters This table details objective measurements from the optic nerve head (ONH), retinal nerve fiber layer (RNFL), and ganglion cell complex (GCC) analyses, comparing the right eye (OD) and left eye (OS). The data reveals a significant overall thinning of the RNFL and GCC layers of the OS compared to the OD (average 7 μm and 14 μm respectively), with all p-values less than 5%, indicating statistically significant findings. This significant inter-eye asymmetry provided the quantitative evidence of optic nerve involvement.

Metric	Unit	OD (Right Eye) Value	OS (Left Eye) Value	Diff. (OD-OS)	p-value
ONH Analysis					
Disc Area	mm^2^	1.80	2.12	0.31	-
Cup Area	mm^2^	0.21	0.67	0.46	-
C/D Area Ratio	-	0.12	0.32	0.2	-
C/D V. Ratio	-	0.38	0.60	0.22	-
RNFL Analysis					
Avg RNFL μm	μm	70	63	-7	< 5%
Inf RNFL μm	μm	74	65	-9	< 5%
GCC Analysis					
Avg GCC μm	μm	86	72	-14	< 5%
Inf GCC μm	μm	93	78	-15	< 5%

Case 3

**Figure 3:**
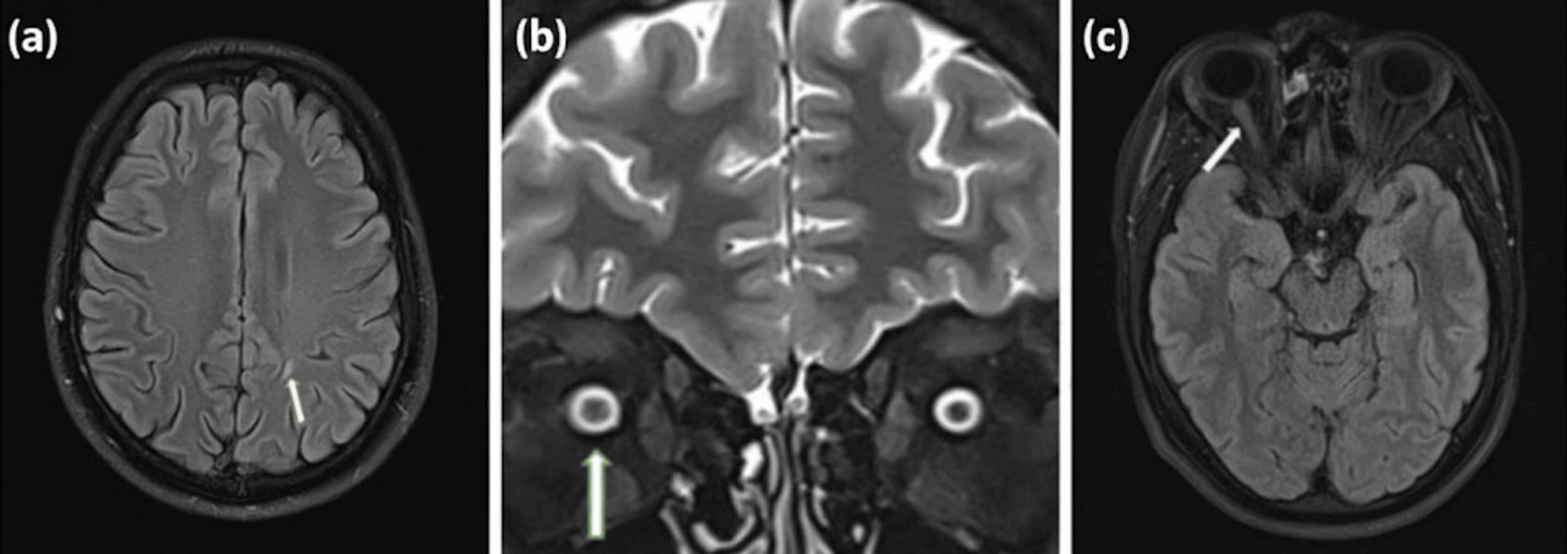
MRI patterns of patient 3 (a): Axial fluid-attenuated inversion recovery (FLAIR) MRI of the brain demonstrating a hyperintense juxtacortical lesion in the right hemisphere (arrow). (b): Coronal T2-weighted orbital MRI showing increased signal intensity and swelling of the right optic nerve (arrow). (c): Axial FLAIR brain MRI demonstrates hyperintensity and mild enlargement of the right optic nerve (arrow), consistent with optic nerve involvement. This combination of a juxtacortical lesion and definitive optic nerve involvement fulfilled the 2024 dissemination in space (DIS) criteria, enabling the multiple sclerosis diagnosis.

A 29-year-old male presented with a one-week history of painless central vision loss in his right eye. Examination revealed decreased visual acuity of 20/30 and mild pallor of the right optic disc. This finding suggested chronic optic nerve atrophy, which was clinically unusual given the acute presentation and hinted at a prior subclinical event. The neurological assessment was otherwise unremarkable. Orbital and brain MRI revealed right optic nerve involvement (edema and increased signal intensity on T2-weighted and fluid-attenuated inversion recovery (FLAIR) sequences) and a well-circumscribed juxtacortical lesion consistent with demyelination (Figure 3). VEPs showed delayed latency on the right eye (125 ms) as compared to the left (105 ms). CSF analysis revealed an elevated IgG index and CSF-restricted OCBs.

Across all three patients, multimodal assessment of the optic nerve was the essential factor in fulfilling the 2024 McDonald criteria for DIS. Under previous criteria, these diagnoses would have been delayed, as each patient presented with only a single demyelinating lesion on brain or spinal MRI (Case 1: periventricular; Case 2: cervical; Case 3: juxtacortical). This solitary lesion was insufficient to establish DIS. However, the 2024 revision, by formally recognizing the optic nerve as a fifth anatomical site, allowed objective evidence of optic nerve involvement to serve as the second DIS location. This was demonstrated using VEPs (Case 1), OCT (Case 2), and orbital MRI (Case 3). In all three patients, DIT was unequivocally established by the presence of CSF-specific OCBs. Following these definitive diagnoses, all three patients were counseled and initiated on appropriate disease-modifying therapy (DMT). These cases therefore provide a direct practical demonstration of how the 2024 criteria can expedite an MS diagnosis and facilitate timely treatment initiation, particularly in patients with subtle clinical histories or limited CNS lesion burden on imaging.

## Discussion

Diagnostic implications of optic nerve involvement in MS

These three cases demonstrated how imaging and electrophysiological studies can reveal subtle signs that support dissemination in the CNS, expediting the diagnosis and treatment of MS. The 2024 McDonald criteria have notably enhanced diagnostic accuracy by designating the optic nerve as a distinct site of DIS, thereby facilitating earlier diagnosis and intervention [9]. Findings support the significant frequency of subclinical involvement of the optic nerve in people with MS, even in the context of no prior visual complaints or a history of optic neuritis [8]. Demonstration of subclinical optic nerve damage in the setting of clinically isolated syndrome (CIS) and early MS has been linked to an increased disease burden [10]. Neurodegenerative retinal changes may be present even in the earliest phases, highlighting the need to prevent disability accumulation in these cases [10].

Early diagnosis and therapeutic implications

Even before the 2024 revisions, evidence supported the benefits of early treatment, now facilitated by recognizing the optic nerve as a fifth DIS site. Trials such as the ONTT, studies in radiologically isolated syndrome (RIS), and pivotal trials of high-efficacy DMTs like natalizumab (AFFIRM and real-world studies) showed that early therapy is expected to improve long-term outcomes, reduce disability progression, and delay clinically definite MS [11]. The ongoing DELIVER-MS study continues to evaluate early high-efficacy therapy in prognosis [11]. With the updated criteria, patients with isolated optic nerve involvement can be diagnosed promptly and started on DMTs earlier, improving both short- and long-term functional outcomes [12].

Multimodal assessment of optic nerve pathology

The optic nerve’s accessibility to multimodal metrics - OCT, orbital MRI, and VEPs - is central to the new criteria and, as our cases show, these tools are highly complementary. OCT is a non-invasive, fast, and reproducible exam to measure the pRNFL and the GCIPL. These metrics, used in Case 2, can differentiate healthy controls from people with MS and quantify neurodegeneration [7]. Thinning of pRNFL and GCIPL correlates with total brain atrophy, white matter changes, cognitive decline, and physical disability, supporting their role as CNS degeneration biomarkers [13]. Complementing OCT, orbital MRI (used in Case 3) allows direct visualization of inflammation, demyelination, and chronic atrophy. High-resolution fat-suppressed T2/STIR and post-contrast T1-weighted images enable recognition of acute lesions, which subsequently progress to measurable atrophy [14]. Advanced sequences can also reveal subclinical lesions in eyes without prior optic neuritis, quantifying cumulative injury [12]. Finally, VEPs (used in Case 1) assess visual pathway function, revealing demyelination and remyelination. Together with OCT and MRI, they provide a comprehensive evaluation of neuroaxonal injury and repair in MS [13].

Future directions and neuroprotection

The quantitative data derived from the optic nerve fosters innovation. Artificial intelligence (AI) models applied to OCT and MRI may help detect subtle changes and inform predictions about conversion or relapse [15]. This data can be combined with blood biomarkers, such as serum neurofilament light chains (NfLs), to create multimodal models for long-term prognostic estimates [6]. Building on this, digital biomarkers and phenotyping - using personal devices to quantify behaviour and physiology in real-time (e.g., eye-tracking, gait) - extend monitoring into patients’ daily environments, offering a path toward continuous, patient-centered surveillance [16]. Beyond diagnosis, the optic nerve also provides a model to explore neuroprotective and remyelination-targeted interventions. Clinical trials have offered limited evidence of remyelination with agents such as clemastine [17], while other trials (e.g., Phase II ACUITY) are evaluating neuroprotective agents to preserve retinal structure after acute optic neuritis [6,18].

Limitations, accessibility and diagnostic precision

Despite these advancements, several limitations remain. As was necessary in all three of our cases, the increased sensitivity is counterbalanced by the need for stringent exclusion of disease mimics such as neuromyelitis optica spectrum disorder (NMOSD) and myelin oligodendrocyte glycoprotein antibody disease (MOGAD). Clinicians must be vigilant for "red flag" presentations, such as bilateral simultaneous ON, severe vision loss, or poor corticosteroid responsiveness, which should prompt serological testing for AQP4-IgG and MOG-IgG antibodies [12]. Imaging can aid differentiation, as MS-related ON lesions tend to be short-segment and unilateral, whereas NMOSD and MOGAD often show longitudinally extensive lesions [13].

Another important limitation concerns the assessment of optic nerve involvement during the acute phase using OCT. Acute inflammatory changes can cause optic disc swelling and transient thickening of the pRNFL, obscuring underlying neuroaxonal loss [19]. This was a relevant consideration in Case 3, who presented with acute vision loss. As demonstrated in that case, this restriction can be overcome by assessing patients in the acute phase by VEPs, which remain highly sensitive [19].

Moreover, our report highlights that accurate demonstration of DIS via the optic nerve frequently requires a combination of methods-integrating high-resolution fat-saturated orbital MRI, OCT, and VEPs. This dependence on specialized equipment and trained neuro-ophthalmology expertise potentially limits accessibility and standardization across clinical settings [12]. The variation in availability of resources also raises concerns over the equitable implementation across health care systems. Concurrently, while the 2024 criteria improve sensitivity, careful application is mandated to balance increased early diagnosis against the risk of overdiagnosis or misdiagnosis, which remains a significant concern [20].

## Conclusions

The revised McDonald Criteria of 2024 has recognized optic nerve as a fifth site for DIS and represent a great advancement; The change in the criteria allows for an earlier and more accurate and timely identification of the disease especially when patients present with atypical or subclinical manifestations. Our cases demonstrate that this change facilitates earlier access to disease-modifying therapies, which is expected to improve long-term patient outcomes.

While our findings are illustrative, this is a small case series, and broader validation in larger, diverse cohorts is necessary to support widespread implementation. Realizing the full benefit of these criteria will require careful application, including the active exclusion of possible mimics. Furthermore, effective implementation depends on managing the technical requirements of multimodal assessment and ensuring equitable access to these advanced neuro-ophthalmology technologies.
